# Supplementary material for: DNA double-strand break-free CRISPR interference delays Huntington’s disease progression in mice
Source: Commun Biol. 2023 Apr 28;6:466. doi: 10.1038/s42003-023-04829-8 (PMC10147674; doi:10.1038/s42003-023-04829-8)
Supplement: Supplementary file 2 — Supplementary Information [file 42003_2023_4829_MOESM2_ESM.docx]

**Supplementary Information**

**
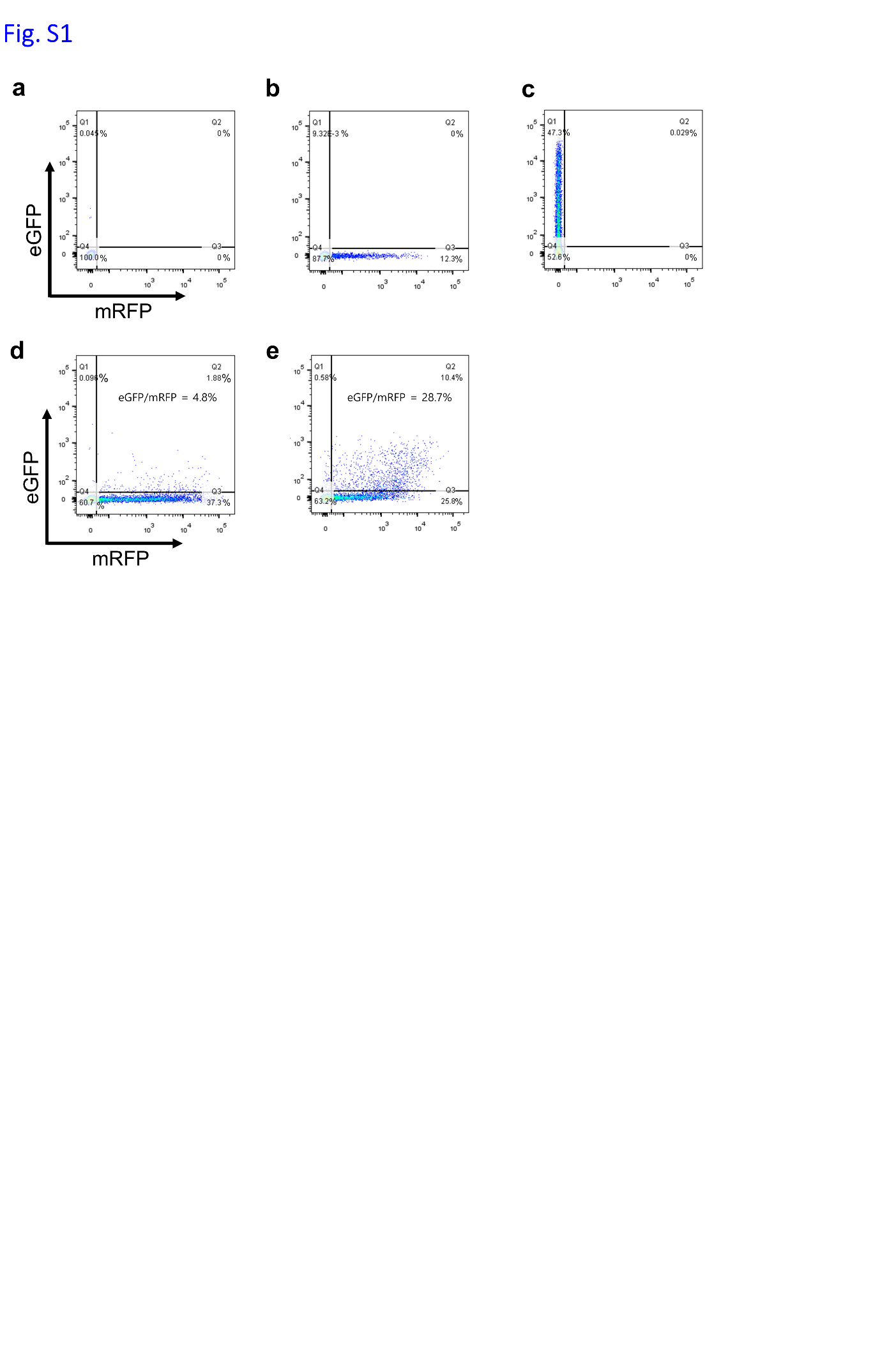
**

**Supplementary Figure 1.** **Analysis of Cas9 efficacy using flow cytometric in transfected HEK293T cells. a-e**, Flow cytometric analysis in transfected HEK293T cells. **a-c**, Non-transfected cells and cells transfected with plasmids expressing eGFP or mRFP alone were used as controls for voltage setting. **d**, Cells transfected with the surrogate reporter alone were used as the negative control. **e**, The efficiency of CAG-targeting sgRNA measured by co-transfection of a plasmid encoding both Cas9 and CAG-targeting sgRNA and the surrogate reporter. The percentage of eGFP^+^ cells in the total mRFP^+^ cell population is shown (e.g., eGFP/mRFP = 28.7%). The percentage of each quadrant is shown.


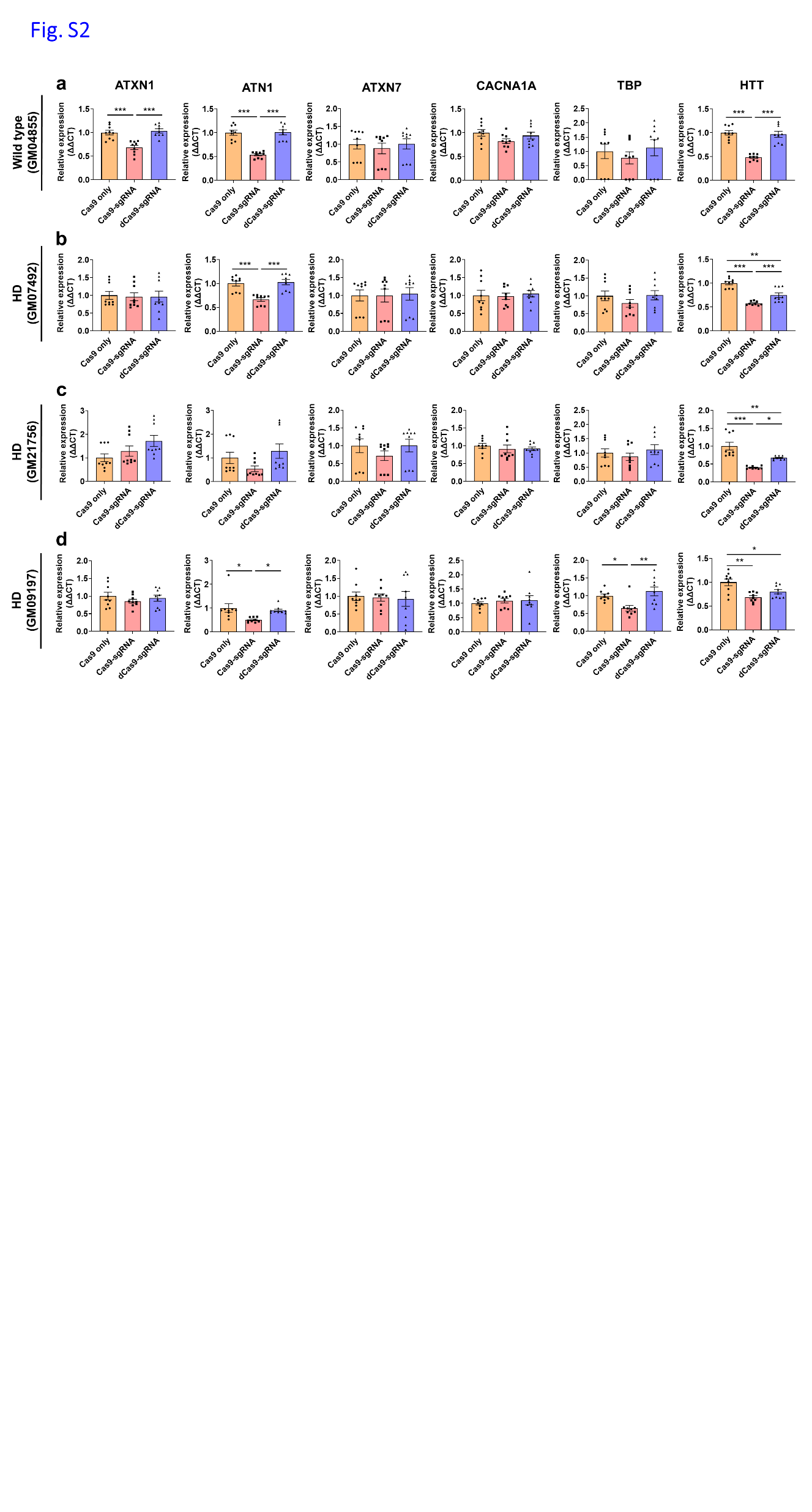


**Supplementary Figure 2. CRISPRi has no effect on genes with CAG repeats in human HD fibroblasts, not in *HTT* gene. a**, In human control fibroblasts, after Cas9-sgRNA treatment, the expression of *ATXN1, ATN1*, and *HTT* genes significantly decreased compared to the Cas9 only- and dCas9-sgRNA treatment. **b**, In HD fibroblast containing 40-50 CAG, *ATN1* and *HTT* were significantly suppressed in the Cas9-sgRNA-treated group compared to the other groups and *HTT* gene expression was decreased in the dCas9-sgRNA group than Cas9 only group. **c**, The Cas9-sgRNA and dCas9-sgRNA significantly reduced the *HTT* gene in GM21756 cell line (HD fibroblasts) compared to Cas9 only, and treatment with Cas9-sgRNA suppressed *HTT* gene expression more than treatment with dCas9-sgRNA. **d**, In GM09197 fibroblast, expression of *ATN1* and *TBP* genes was significantly suppressed by the Cas9-sgRNA treatment and *HTT* gene expression was significantly decreased in both the Cas9-sgRNA group and dCas9-sgRNA group compared to Cas9 only group (n = 3, each). Error bars represent the mean ± S.E.M. **P*< 0.05, ***P*< 0.01, and ****P*< 0.001, by one-way ANOVA with Bonferroni comparison.

**
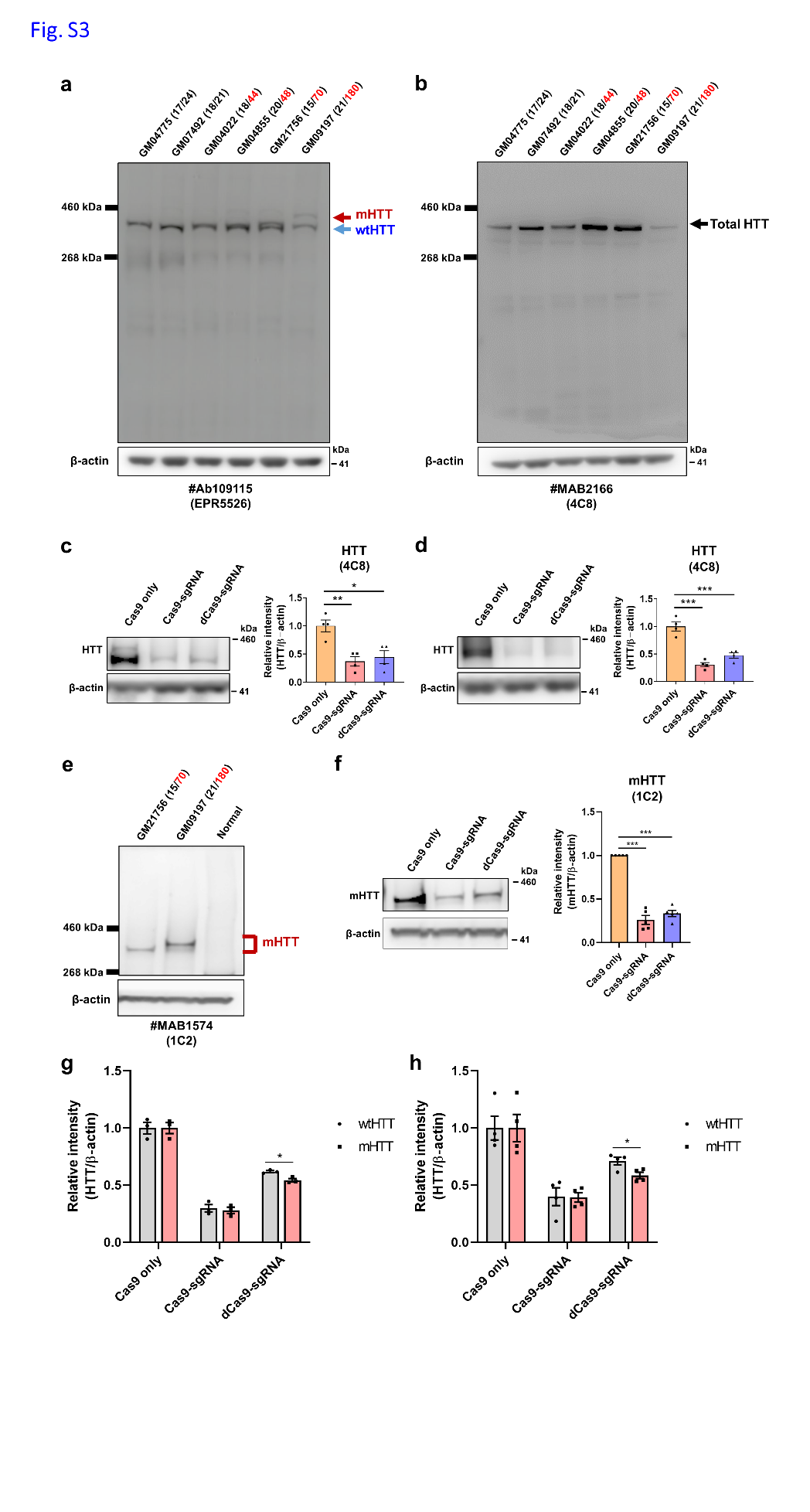
**

**Supplementary Figure 3. Expression of wtHTT and mHTT in human HD fibroblast lines. a**,**b**, Western blot analysis of wtHTT and mHTT expression in human HD fibroblast lines. The ratio of CAG repeats in the wtHTT versus mHTT alleles in the three human HD fibroblast lines is as follows: 18/44 (GM04022), 20/48 (GM04855),15/70 (GM21756), and 21/180 (GM09197); and normal fibroblast is as follows: 17/24 (GM04775) and 18/21 (GM07492). Whole cell lysates of the HD fibroblasts were analyzed by Western blot with Ab109115 (clone name, EPR5526) and MAB2166 (clone name, 4C8) antibodies. **a**, EPR5526 antibody can distinguishable wtHTT (lower band) and mHTT (upper band) in GM09197 and GM21756 cell lysates. **b**, The total HTT was showed by MAB2166 (clone name, 4C8) antibody. **c**, Representative Western blot images by 4C8 antibody in GM09197 fibroblast (left panel). Total HTT protein expression was significantly reduced by both Cas9-sgRNA and dCas9-sgRNA compared with Cas9 alone (n = 4, each). **d**, Representative Western blot images by 4C8 antibody in GM09197 fibroblast (left panel). Total HTT protein expression was significantly reduced by both Cas9-sgRNA and dCas9-sgRNA compared with Cas9 alone (n = 4, each). **e**, The MAB1574 antibody revealed only mHTT bands (polyglutamine region) below each lane. **f**, Western blot images showing mHTT levels after treatment with Cas9-sgRNA, dCas9-sgRNA, or Cas9 alone in GM09197 fibroblasts (left panel). When a mHTT antibody (1C2) was used to detect polyglutamine region, expression of the mHTT protein was significantly reduced by both Cas9-sgRNA and dCas9-sgRNA (right panel; n = 5, each). Error bars represent the mean ± S.E.M. **P*< 0.05, ***P*< 0.01, and ****P*< 0.001, by one-way ANOVA with Bonferroni comparison. **g**,**h**, In order to identify the significantly differences of mHTT versus wtHTT protein for the dCas9-sgRNA-treated group, a comparative analysis was performed using the results in Figure 2d and 2e. The mHTT protein was significantly decreased compared to the wtHTT protein in the group treated with dCas9-sgRNA in both GM09197 (**g**) and GM21756 (**h**) fibroblasts (GM09197, n = 3, each; GM21756, n = 4, each). There were no significantly differences in the other groups. Error bars represent the mean ± S.E.M. **P*< 0.05 by independent t-test.


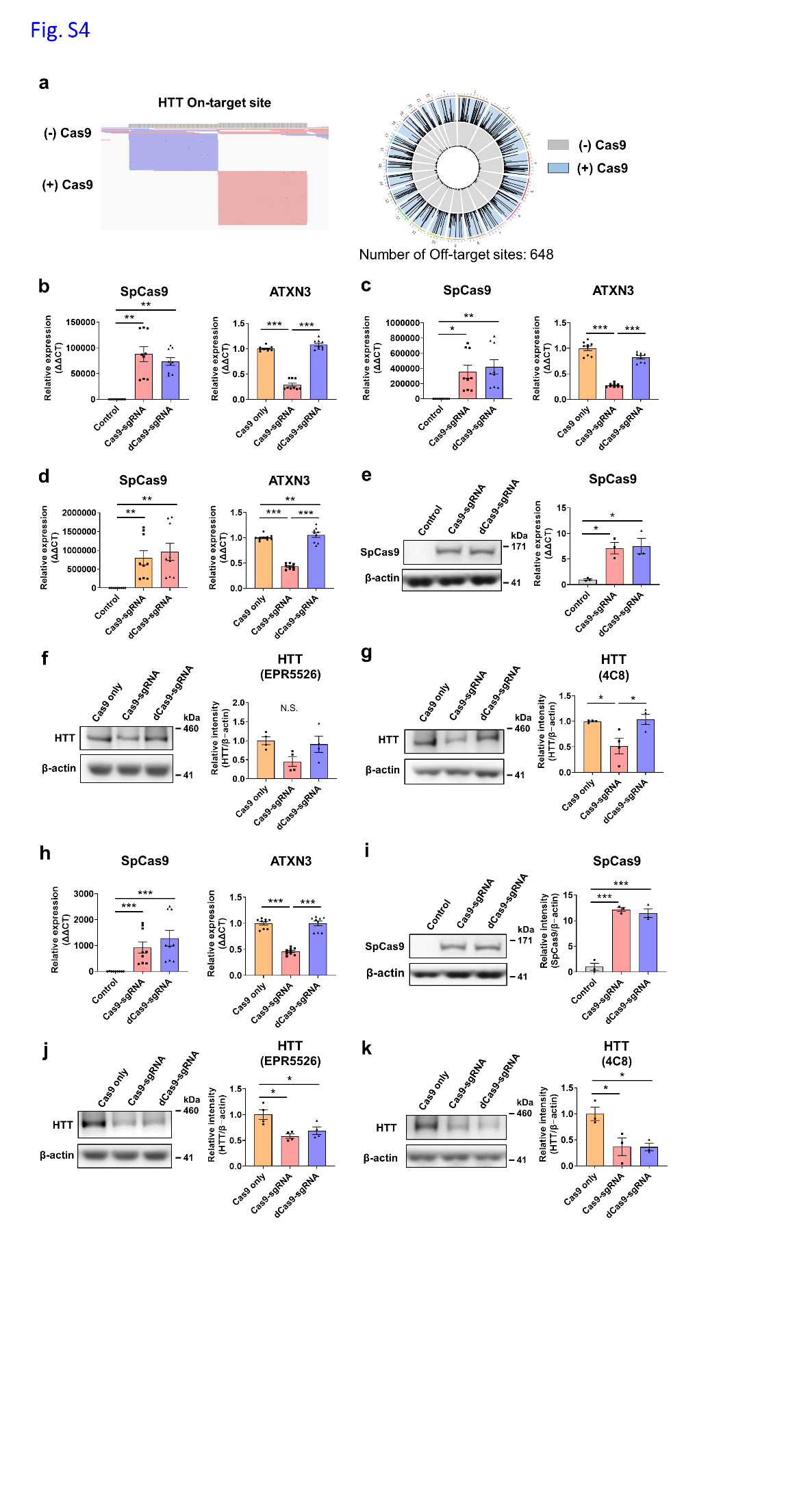


**Supplementary Figure 4. Expression of wtHTT and mHTT in human HD and normal fibroblast lines. a**, Digenome-seq analysis were performed in human HD fibroblast to define off-target effects of CRIPSR-Cas9 nuclease. A representative IGV image showing a staggered alignment and a straight alignment of whole-genome sequence reads at the HTT on-target sites (left panel). Genome-wide Circos plots showing DNA cleavage score of Intact genomic DNA (grey) and Cas9-mediated digested genomic DNA (blue) (right panel). **b,c**, *SpCas9* and *ATXN3* mRNA expression in both GM09197 and GM21756 fibroblasts. *SpCas9* was significantly expressed by Cas9-sgRNA and dCas9-sgRNA in human HD fibroblasts. The expression of *ATXN3* was lower in the Cas9-sgRNA-treated cells compared to the Cas9-sgRNA- and Cas9 only- treated cells (n = 3, each). **d**, In normal fibroblast, mRNA expression of *SpCas9* was stable expressed by Cas9-sgRNA and dCas9-sgRNA. Results of qRT-PCR showing that *ATXN3* was significantly decreased by Cas9-sgRNA compared to the dCas9-sgRNA- and Cas9 only- treated cells (n = 3, each). **e**, Representative Western blot images (left panel, respectively). Quantitative analysis of SpCas9 protein abundance indicated that both Cas9 and dCas9 were stably expressed in normal fibroblasts at 10 days after treatment (n = 3, each). **f**, Western blot images showing HTT levels after treatment with Cas9-sgRNA, dCas9-sgRNA, or Cas9 alone in normal fibroblasts (left panel). When an EPR5526 antibody was used to detect HTT protein, expression of the HTT protein showed a tendency to decrease in the Cas9-sgRNA-treated cells (right panel; n = 4, each). **g**, Representative Western blot images (left panel, respectively). When an 4C8 antibody was used to detect HTT protein, expression of the HTT protein was significantly reduced by Cas9-sgRNA compared to the dCas9-sgRNA- and Cas9 only- treated cells (right panel; n = 4, each). **h**, In HD fibroblast containing 40-50 CAG, mRNA expression of *SpCas9* was stable expressed by Cas9-sgRNA and dCas9-sgRNA. Results of qRT-PCR showing that *ATXN3* was significantly decreased by Cas9-sgRNA compared to the dCas9-sgRNA- and Cas9 only- treated cells (n = 3, each). **i**, Representative Western blot images (left panel, respectively). Quantitative analysis of SpCas9 protein abundance indicated that both Cas9 and dCas9 were stably expressed in HD fibroblast at 10 days after treatment (n = 3, each). **j**, Western blot images showing HTT levels after treatment with Cas9-sgRNA, dCas9-sgRNA, or Cas9 alone in HD fibroblast containing 40-50 CAG (left panel). When an EPR5526 antibody was used to detect huntingtin protein, expression of the HTT protein was significantly reduced by both Cas9-sgRNA and dCas9-sgRNA (right panel; n = 3, each). **k**, Representative Western blot images (left panel, respectively). When an 4C8 antibody was used to detect huntingtin protein, expression of the HTT protein was significantly reduced by Cas9-sgRNA compared to the dCas9-sgRNA- and Cas9 only- treated cells (right panel; n = 3, each). Error bars represent the mean ± S.E.M. **P*< 0.05, ***P*< 0.01, and ****P*< 0.001, by one-way ANOVA with Bonferroni comparison.


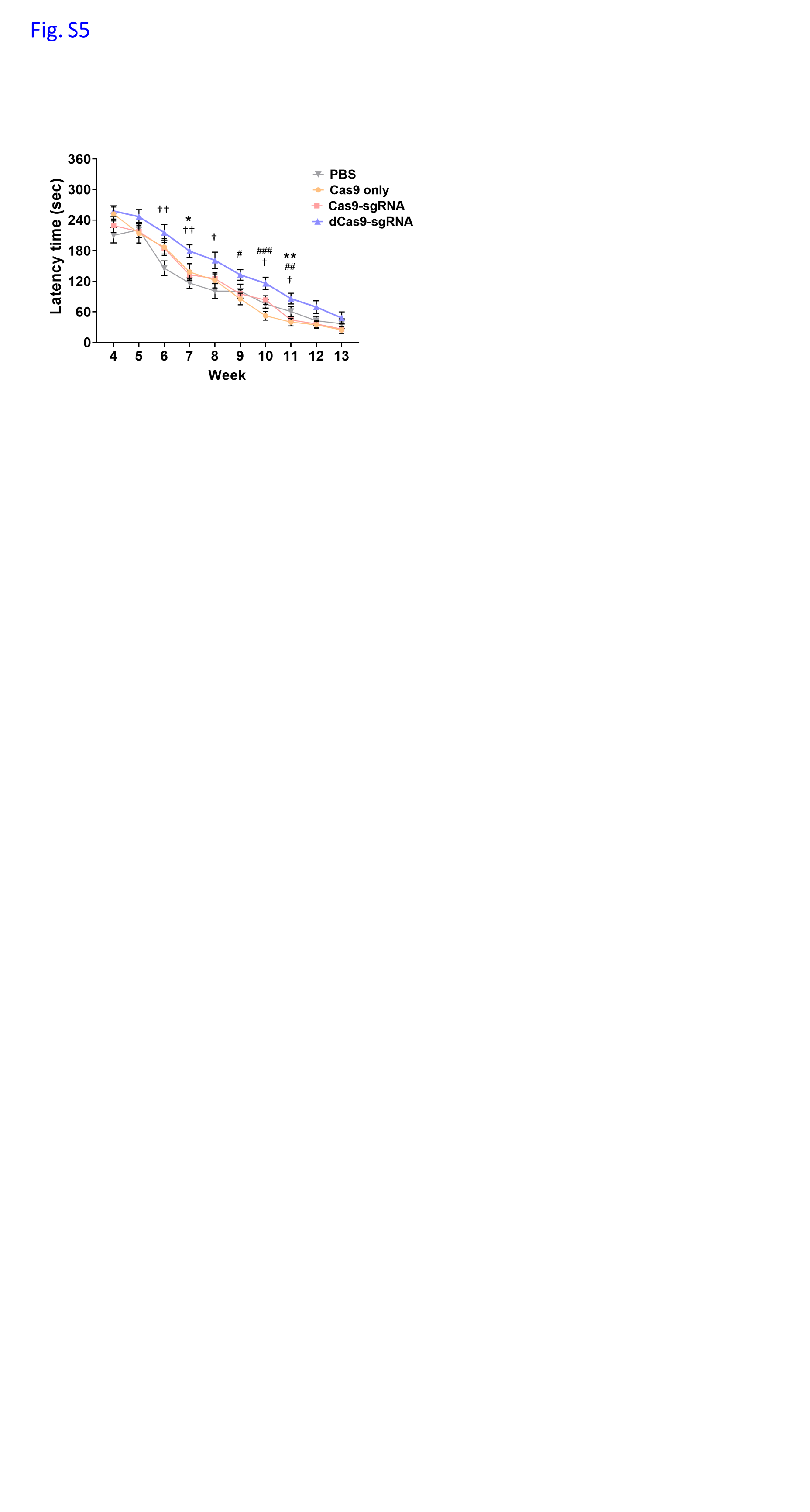


**Supplementary Figure 5. CRISPRi delays disease progression in a mouse model of HD.** Results from a rotarod test at accelerating speed (4-40 rpm). When motor performance was evaluated by the rotarod test, dCas9-sgRNA-treated mice (n = 8) exhibited delayed motor deterioration compared to Cas9-sgRNA-treated mice (n = 14), Cas9 only (n = 8) and PBS control group (n = 11) at two to seven weeks after treatment (at 6-11 weeks of age). Error bars represent the mean ± S.E.M. **P*< 0.05 and ***P*< 0.01, and ****P*< 0.001 versus Cas9-sgRNA; ^##^*P*< 0.01 and ^###^*P*< 0.001 versus Cas9 only; †*P*< 0.05, ††*P*< 0.01, and †††*P*< 0.001, versus PBS, by one-way ANOVA with Bonferroni comparison.


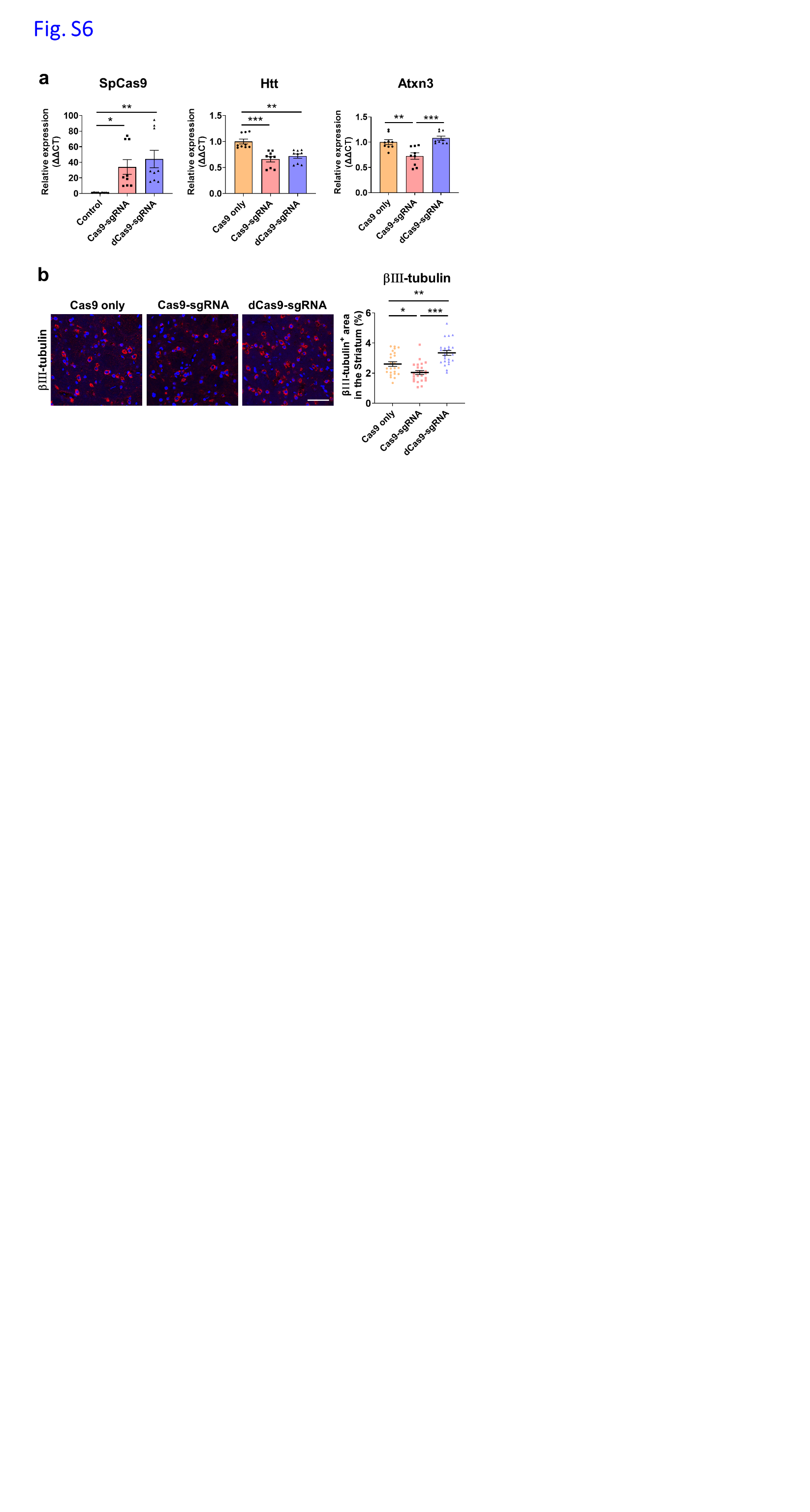


**Supplementary Figure 6. CRISPRi protects striatal neurons in wild-type mice. a**, mRNA expression of genes of interest using qRT-PCR. SpCas9 was significantly expressed by Cas9-sgRNA and dCas9-sgRNA in the striatum of wild-type mice at 4 weeks after treatment (n = 3, each). Htt levels expression was significantly reduced by both Cas9-sgRNA and dCas9-sgRNA compared with Cas9 alone (n = 3, each). Results of qRT-PCR showing that Atxn3 was significantly decreased by Cas9-sgRNA compared to the dCas9-sgRNA- and Cas9 only- treated groups (n = 3, each). **b,** Representative confocal microscopic images of βIII-tubulin^+^ cells in striatal sections from 8-week-old wild-type mice (left panel; scale bars, 50 μm). Quantified results from immunohistochemical analysis (right panel). The densities of βIII-tubulin^+^ was significantly higher in the striatum from dCas9-sgRNA-treated versus Cas9-sgRNA-treated and control mice, and the densities of βIII-tubulin^+^ decreased in Cas9-sgRNA-treated versus dCas9-sgRNA- and Cas9 only-treated mice (n = 3, each). Error bars represent the mean ± S.E.M. **P*< 0.05, ***P*< 0.01, and ****P*< 0.001, by one-way ANOVA with Bonferroni comparison.


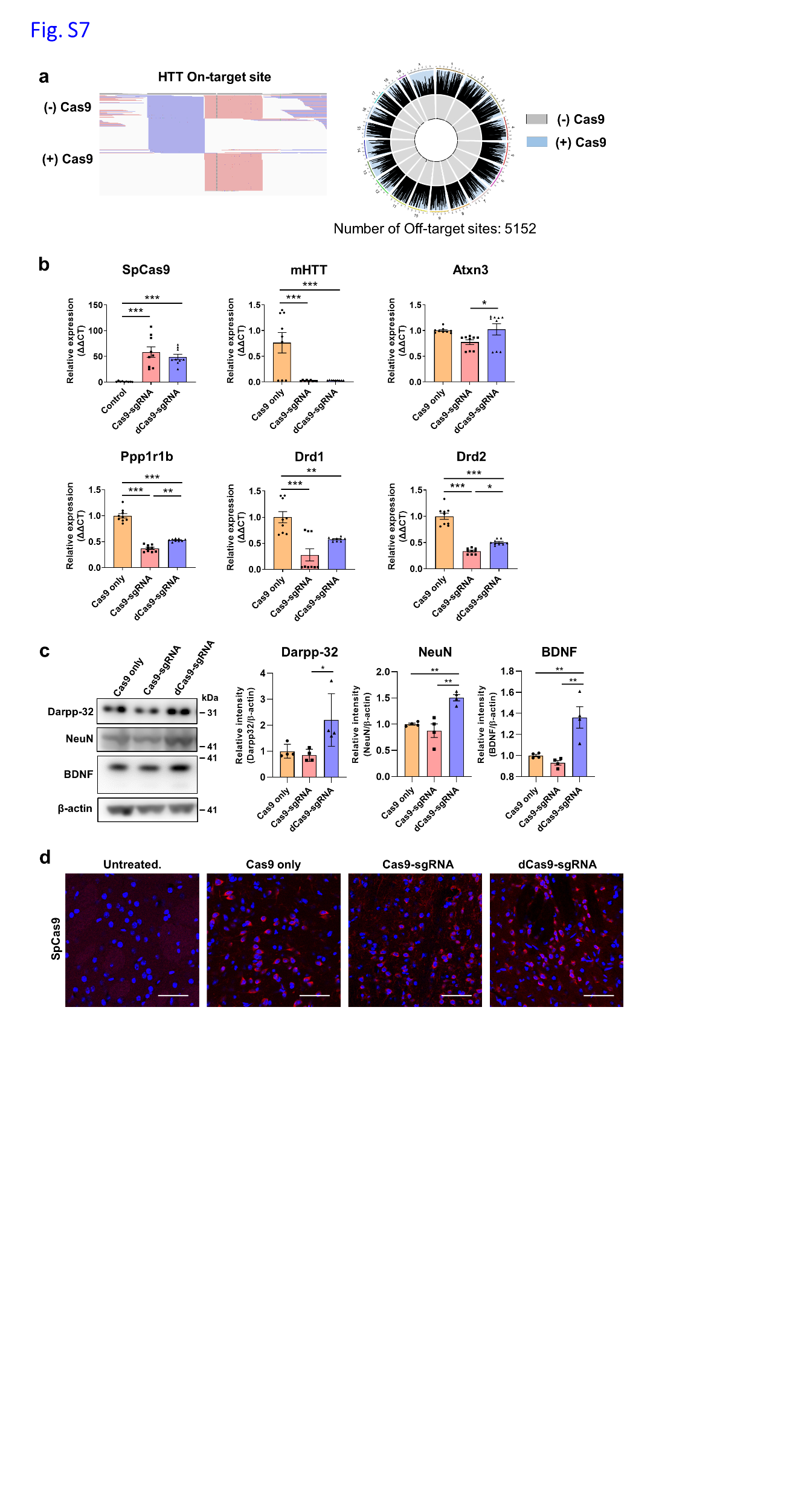


**Supplementary Figure 7. CRISPRi protects striatal neurons in a mouse model of HD. a**, To CRIPSR-Cas9 nuclease off-target DNA cleavage, Digenome-seq analysis were performed in mouse genome. A representative IGV image showing a staggered alignment and a straight alignment of whole-genome sequence reads at the HTT on-target sites (left panel). Genome-wide Circos plots image was showed that mouse genomic DNA (gray) of Cas9-digested genomic DNA (blue) was subjected to whole-genome sequencing (right panel). **b**, mRNA expression of genes of interest using qRT-PCR. *SpCas9* was significantly expressed by Cas9-sgRNA and dCas9-sgRNA in the striatum of R6/2 mice at 4 weeks after treatment (n = 3, each). The *mHTT* levels expression was significantly reduced by both Cas9-sgRNA and dCas9-sgRNA compared with Cas9 alone (n = 3, each). *Atxn3* expression showed significantly decrease in Cas9-sgRNA compared to the dCas9-sgRNA-treated groups (n = 3, each). Error bars represent the mean ± S.E.M. **P*< 0.05, by one-way ANOVA with Tukey comparison. The expression of *Ppp1r1b* and *Drd2* was higher in the dCas9-sgRNA-treated group compared to the Cas9-sgRNA-treated group (n = 3, each). The result of *Drd1* expression showed a significant decrease in both dCas9-sgRNA-treated and Cas9-sgRNA-treated mice. **c,** Images of Western blots probed with antibodies recognizing Darpp-32, NeuN and BDNF in striatal tissue from 8-week-old R6/2 mice. β-actin was used as a loading control (left panel). Quantified results from Western blot analysis (right panel). Darpp-32, NeuN and BDNF levels increased versus Cas9-sgRNA-treated or Cas9-only-treated mice (n = 4, each). Error bars represent the mean ± S.E.M. **P*< 0.05, ***P*< 0.01, and ****P*< 0.001, by one-way ANOVA with Bonferroni comparison. **d,** Representative confocal microscopic images (scale bars, 50 μm). SpCas9^+^ cells were expressed in R6/2 mice brain at 4 weeks after treatment.


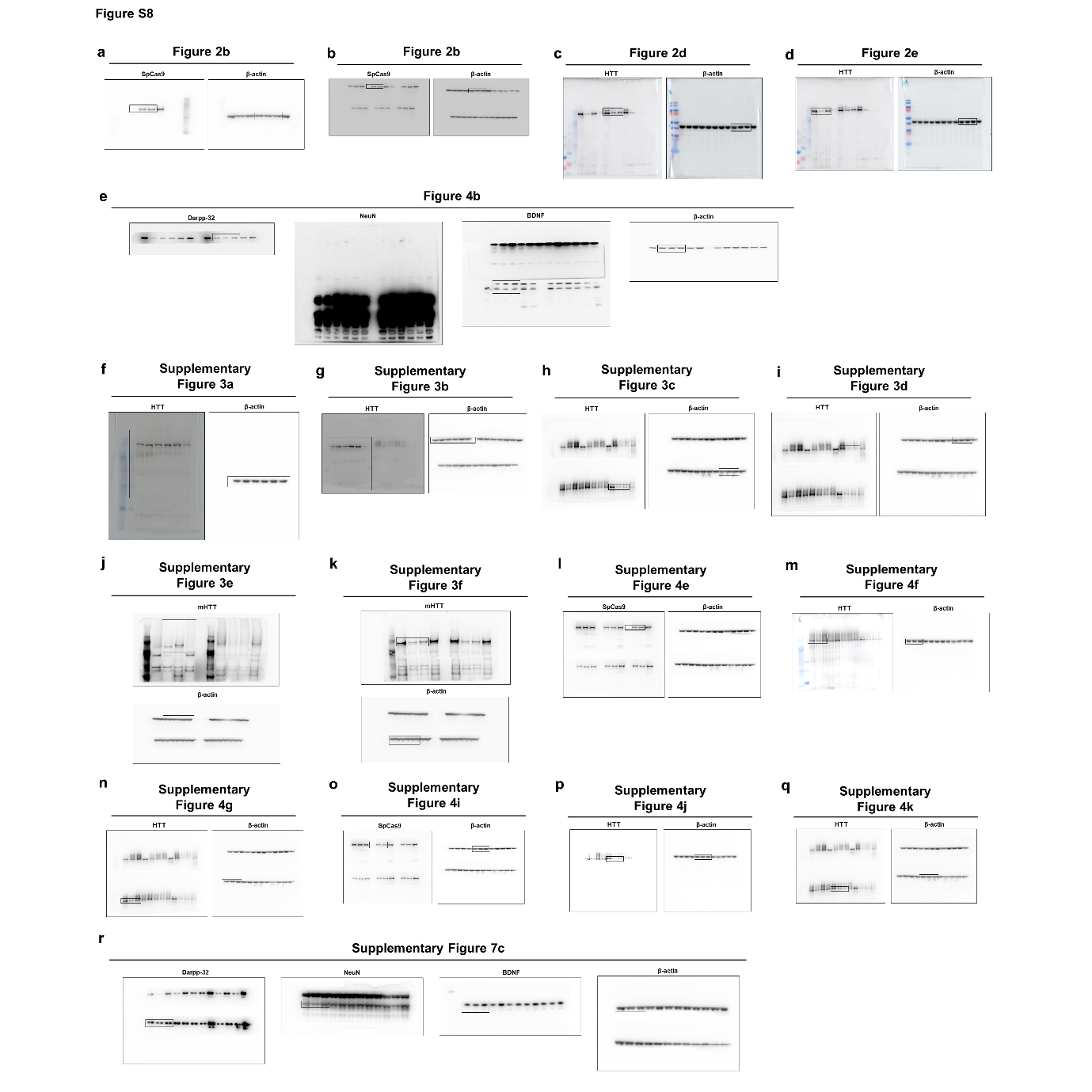


**Supplementary Figure 8. Original uncropped scans for all presented Western blots. a**, corresponds to Figure 2b; **b**, corresponds to Figure 2b; **c**, corresponds to Figure 2c; **d**, corresponds to Figure 2d; **e**, corresponds to Figure 4b; **f**, corresponds to Supplementary Figure 3a; **g**, corresponds to Supplementary Figure 3b; **h**, corresponds to Supplementary Figure 3c; **i**, corresponds to Supplementary Figure 3d; **j**, corresponds to Supplementary Figure 3e; **k**, corresponds to Supplementary Figure 3f; **l**, corresponds to Supplementary Figure 4e; **m**, corresponds to Supplementary Figure 4f; **n**, corresponds to Supplementary Figure 4g; **o**, corresponds to Supplementary Figure 4i; **p**, corresponds to Supplementary Figure 4j; **q**, corresponds to Supplementary Figure 4k; **r**, corresponds to Supplementary Figure 7c.

**Supplementary Table 1.** **The sequence of sgRNA and primers for qRT-PCR.**

| **sgRNA** |  | **Spacer sequence** |
| --- | --- | --- |
| *sgRNA (CAG PAM)* |  | AGCAGCAGCAGCAGCAGCAG |
| **Gene** | **Direction** | **Primer sequence** |
| *SpCas9* | Forward | AAACAGCAGATTCGCCTGGA |
|  | Reverse | TCATCCGCTCGATGAAGCTC |
| **Human gene** | **Direction** | **Primer sequence** |
| *HTT* | Forward | GCACCGACCAAAGAAAGAAC |
|  | Reverse | CCACCATCCTGACATCTGAC |
| *ABHD11* | Forward | CTGACGGTGGATGCTCGTAA |
|  | Reverse | GAGAAAGAGTGTTGGCCCGA |
| *ATXN1* | Forward | CCGGAGCCCTGCTGAGGT |
|  | Reverse | CCAGACGCCGGGACAC |
| *ATN1* | Forward | ACCTGTACTTCGTGCCACTG |
|  | Reverse | TTGCGATTGCCAGGAGACAT |
| *ATXN3* | Forward | GAGCTTCGGAAGAGACGAGA |
|  | Reverse | GATCACTCCCAAGTGCTCCT |
| *ATXN7* | Forward | GGGAAAAACCGCGAAGTCAT |
|  | Reverse | CGGCTTGCTGGATGAGCTA |
| *CACNA1A* | Forward | AGACCCCCGAGAATAGCCTT |
|  | Reverse | GGTCTGGGTTGGCGTTTTTG |
| *DCP1B* | Forward | GTGGCTCTGTACACCTTCGG |
|  | Reverse | CCCCCATAGCCACTGATACC |
| *FOXJ2* | Forward | ACACTGGCTTTGCCTTTCCT |
|  | Reverse | TGGGTACATTGGTGATGGGC |
| *FZD1* | Forward | AGACCATCATGCCCAACCTG |
|  | Reverse | CAGAACTCTGGAAGCAGCGA |
| *KMT2D* | Forward | CTTCCCTAGCGATGACCGAC |
|  | Reverse | TCAATGAACTGGGCAGGACC |
| *PLEC* | Forward | ACCAAGTGGGTCAACAAGCA |
|  | Reverse | CTTCTCCTTGGCCGTCATGT |
| *SATB1* | Forward | CGGGCCATCTGATGAAAAC |
|  | Reverse | CATATCCTTTCTCACCAGCAC |
| *TBP* | Forward | GACCCCACAGCCTATTCAGA |
|  | Reverse | TTGACTGCTGAACGGCTGCA |
| *ZNF384* | Forward | TGTCCTCTCCCCTGAGGATG |
|  | Reverse | CGTCTGTGGGACTGCAGATT |
| *ZNF395* | Forward | CTTTCACCACCTCTGATGAC |
|  | Reverse | CCACACATAAACCTTCTGCC |
| *ZNF853* | Forward | AGCACTCGAATCTGGTGACG |
|  | Reverse | GTGGCTGTAGCGGCCTG |
| *GAPDH* | Forward | AAGGGTCATCATCTCTGCCC |
|  | Reverse | GTGAGTGCATGGACTGTGGT |
| **Mouse gene** | **Direction** | **Primer sequence** |
| *mHTT* | Forward | AGGTTCGCTTTTACCTGCGG |
|  | Reverse | CATCAGCTTTTCCAGGGTCG |
| *Atxn3* | Forward | AGGCAAGCAGTGGTTTAACT |
|  | Reverse | GGCAGATCACCCTTAACAACA |
| *Ppp1r1b* | Forward | AGATTCAGTTCTCTGTGCCCG |
|  | Reverse | TGGGTCTCTTCGACTTTGGG |
| *Drd1* | Forward | TGGAAACCCTGTCGAATGCT |
|  | Reverse | TCAATGCAGAATGGCTGGGT |
| *Drd2* | Forward | AGTGAACAGGCGGAGAATGG |
|  | Reverse | TAGACCGTGGTGGGATGGAT |
| *Gapdh* | Forward | CAAGGTCATCCATGACAACTTTG |
|  | Reverse | GTCCACCACCCTGTTGCTGTAG |

**Supplementary Table 2.** **The antibodies for Western blot (WB) and immunohistochemistry (IHC).**

| Antibodies | Cat. No. | Lot. No. | Source | Dilution |
| --- | --- | --- | --- | --- |
| SpCas9 | Ab191468 | GR3189443-2 | Abcam | WB, 1:1000; IHC, 1:400 |
| Polyglutamine expansion (1C2) | Mab1574 | 3071582 | Millipore | WB, 1:1000 |
| Huntingtin (4C8) | Mab2166 | 3850451 | Millipore | WB, 1:1000 |
| Huntingtin (mEM48) | Mab5374 | 2796369 | Millipore | IHC, 1:400 |
| Huntingtin (EPR5526) | Ab109115 | GR3187604-2 | Abcam | WB, 1:1000 |
| DARPP-32 | 2306S | 7 | Cell Signaling Technology | WB, 1:1000 |
| NeuN | Mab377 | 324801 | Millipore | WB, 1:2000; IHC, 1:200 |
| βIII-tubulin | 801201 | B264428 | BioLegend | IHC, 1:400 |
| BDNF | Ab108319 | GR3227037-4 | Abcam | WB, 1:1000; IHC, 1:200 |
| β-actin | SC-47778 | C1919 | Santa Cruz Biotechnology | WB, 1:2000 |
| Mouse IgGk BP-HRP | SC-516102 | B0519 | Santa Cruz Biotechnology | WB, 1:3000 |
| Rabbit IgG-HRP | SC-2357 | L1218 | Santa Cruz Biotechnology | WB, 1:3000 |
| Rabbit IgG (H+L), Alexa Flor 594 | Ab150080 | GR3232361 | Invitrogen | IHC, 1:400 |
| Mouse IgG (H+L), Alexa Flor 594 | A11005 | 2043369 | Invitrogen | IHC, 1:400 |
| Rabbit IgG (H+L), Alexa Flor 488 | A11001 | 2140660 | Invitrogen | IHC, 1:400 |
| Mouse IgG (H+L), Alexa Flor 488 | A11008 | 2051237 | Invitrogen | IHC, 1:400 |
