# Supplementary material for: DNA double-strand break-free CRISPR interference delays Huntington’s disease progression in mice
Source: Commun Biol. 2023 Apr 28;6:466. doi: 10.1038/s42003-023-04829-8 (PMC10147674; doi:10.1038/s42003-023-04829-8)
Supplement: Supplementary file 3 — Description of Additional Supplementary Files [file 42003_2023_4829_MOESM3_ESM.docx]

Description of Additional Supplementary Files

**File name:** Supplementary Data 1

**Description:** Potential off-target sites for sgRNA targeting CAG repeat region in HTT gene via OFFinder algorithm in human genome

**File name:** Supplementary Data 2

**Description:** The source data for plotting all the graphs in the supplementary file

**File name:** Supplementary Data 3

**Description:** Potential off-target sites in the RNA sequencing of the R6/2 brain
