# Supplementary material for: DNA double-strand break-free CRISPR interference delays Huntington’s disease progression in mice
Source: Commun Biol. 2023 Apr 28;6:466. doi: 10.1038/s42003-023-04829-8 (PMC10147674; doi:10.1038/s42003-023-04829-8)
Supplement: Supplementary file 6 — Supplementary Data 3 [file 42003_2023_4829_MOESM6_ESM.docx]

**Supplementary Data 3. Potential off-target sites in the RNA sequencing of the R6/2 brain.**

| **Gene_ID** | **Transcript_ID** | **Gene_Symbol** | **Description** | **Fold change** |
| --- | --- | --- | --- | --- |
| 11602 | NM_009641 | ANGPT4 | angiopoietin 4 | 1.022605 |
| 11682 | NM_007439 | ALK | anaplastic lymphoma kinase | 1.141711 |
| 11819 | NM_009697,NM_183261 | NR2F2 | nuclear receptor subfamily 2, group F, member 2 | -1.366097 |
| 11881 | NM_009712 | ARSB | arylsulfatase B | 1.026157 |
| 11906 | NM_007496 | ZFHX3 | zinc finger homeobox 3 | -1.143772 |
| 11991 | NM_001077265,NM_001077266,NM_001077267,NM_007516 | HNRNPD | heterogeneous nuclear ribonucleoprotein D | -1.291417 |
| 12211 | NM_007566 | BIRC6 | baculoviral IAP repeat-containing 6 | -1.006049 |
| 12296 | NM_001252533,NM_001309519,NM_023116 | CACNB2 | calcium channel, voltage-dependent, beta 2 subunit | -1.015517 |
| 13040 | NM_001267695,NM_021281 | CTSS | cathepsin S | -1.556789 |
| 13134 | NM_001038610,NM_007826 | DACH1 | dachshund 1 (Drosophila) | -1.262284 |
| 13653 | NM_007913 | EGR1 | early growth response 1 | -1.518985 |
| 13804 | NM_007931 | ENDOG | endonuclease G | 1.301255 |
| 13998 | NM_053072 | FGD6 | FYVE, RhoGEF and PH domain containing 6 | 1.032911 |
| 14011 | NM_001303102,NM_007961 | ETV6 | ets variant 6 | -1.145630 |
| 14183 | NM_001347638,NM_010207,NM_201601 | FGFR2 | fibroblast growth factor receptor 2 | -1.167678 |
| 14254 | NM_010228 | FLT1 | FMS-like tyrosine kinase 1 | -1.161472 |
| 14451 | NM_008086 | GAS1 | growth arrest specific 1 | -1.130427 |
| 14660 | NM_001081081,NM_001113383 | GLS | glutaminase | -1.092162 |
| 16009 | NM_008343 | IGFBP3 | insulin-like growth factor binding protein 3 | 1.059974 |
| 16319 | NM_016692 | INCENP | inner centromere protein | -1.121911 |
| 16512 | NM_010601 | KCNH3 | potassium voltage-gated channel, subfamily H (eag-related), member 3 | -1.282151 |
| 16531 | NM_001253358,NM_001253359,NM_001253360,NM_001253361,NM_001253362,NM_001253363,NM_001253364,NM_001253365,NM_001253366,NM_001253367,NM_001253368,NM_001253369,NM_001253370,NM_001253371,NM_001253372,NM_001253373,NM_001253374,NM_001253375,NM_001253376,NM_001253377,NM_001253378,NM_010610 | KCNMA1 | potassium large conductance calcium-activated channel, subfamily M, alpha member 1 | -1.019423 |
| 16651 | NM_001310837,NM_010656 | SSPN | sarcospan | -1.052433 |
| 16764 | NM_001290814 | AFF3 | AF4/FMR2 family, member 3 | 1.012657 |
| 17289 | NM_008587 | MERTK | c-mer proto-oncogene tyrosine kinase | -1.023510 |
| 18015 | NM_010897 | NF1 | neurofibromatosis 1 | -1.143017 |
| 18115 | NM_001308506,NM_008710 | NNT | nicotinamide nucleotide transhydrogenase | -1.071399 |
| 18191 | NM_001198587,NM_001252074,NM_172544 | NRXN3 | neurexin III | 1.075645 |
| 19206 | NM_001328514,NM_008957 | PTCH1 | patched 1 | 1.027512 |
| 19883 | NM_001289916,NM_001289917,NM_013646 | RORA | RAR-related orphan receptor alpha | -1.613458 |
| 20616 | NM_001277982,NM_001277983,NM_001277985,NM_001277986,NM_013669,NR_102729 | SNAP91 | synaptosomal-associated protein 91 | -1.202735 |
| 20649 | NM_016667 | SNTB1 | syntrophin, basic 1 | 1.141598 |
| 20666 | NM_009234 | SOX11 | SRY (sex determining region Y)-box 11 | -1.290839 |
| 20855 | NM_009285 | STC1 | stanniocalcin 1 | 1.081699 |
| 21411 | NM_001114140,NM_013836 | TCF20 | transcription factor 20 | -1.034940 |
| 22349 | NM_009509 | VIL1 | villin 1 | -1.007086 |
| 22589 | NM_009530 | ATRX | alpha thalassemia/mental retardation syndrome X-linked | -1.331414 |
| 22590 | NM_011728 | XPA | xeroderma pigmentosum, complementation group A | 1.054924 |
| 22772 | NM_009574 | ZIC2 | zinc finger protein of the cerebellum 2 | -1.023779 |
| 23836 | NM_011800 | CDH20 | cadherin 20 | 1.054006 |
| 26903 | NM_001077694,NM_001310152,NM_021469 | DYSF | dysferlin | 1.050714 |
| 27410 | NM_001039581,NM_013855 | ABCA3 | ATP-binding cassette, sub-family A (ABC1), member 3 | 1.176224 |
| 29819 | NM_001111272,NM_001347044,NM_001347045,NM_025303 | STAU2 | staufen (RNA binding protein) homolog 2 (Drosophila) | -1.099172 |
| 50754 | NM_001177773,NM_001177774,NM_080428 | FBXW7 | F-box and WD-40 domain protein 7 | -1.364758 |
| 50914 | NM_016968 | OLIG1 | oligodendrocyte transcription factor 1 | 1.082750 |
| 50932 | NM_001045959,NM_001045964,NM_016713,NM_176893 | MINK1 | misshapen-like kinase 1 (zebrafish) | -1.048845 |
| 52372 | NM_001167937,NM_001167938 | D6Ertd527e | DNA segment, Chr 6, ERATO Doi 527, expressed | 1.000698 |
| 53761 | NM_001199044,NM_020027 | PRRC2A | proline-rich coiled-coil 2A | -1.269542 |
| 54367 | NM_018759,NR_030777 | ZFP326 | zinc finger protein 326 | -1.192049 |
| 54561 | NM_138742 | NAP1l3 | nucleosome assembly protein 1-like 3 | -1.186508 |
| 54636 | NM_001290792,NM_001290794,NM_001290795,NM_172372 | WDR45 | WD repeat domain 45 | 1.241612 |
| 54650 | NM_001166531,NM_001166532,NM_019460 | SFMBT1 | Scm-like with four mbt domains 1 | -1.104738 |
| 54720 | NM_001081549,NM_019466 | RCAN1 | regulator of calcineurin 1 | 1.222372 |
| 56541 | NM_019986 | HABP4 | hyaluronic acid binding protein 4 | 1.038640 |
| 58194 | NM_001135727,NM_001135728,NM_001290661,NM_001290664,NM_021389,NR_110969,NR_110970 | SH3KBP1 | SH3-domain kinase binding protein 1 | -1.101286 |
| 63955 | NM_001146287,NM_022021 | CABLES1 | CDK5 and Abl enzyme substrate 1 | 1.186458 |
| 63958 | NM_022022 | UBE4B | ubiquitination factor E4B | -1.108331 |
| 65973 | NM_001177849,NM_001177850,NM_001177851,NM_001177852,NM_001177853,NM_001177854,NM_001177855,NM_001177856,NM_001290367,NM_023066,NM_133723 | ASPH | aspartate-beta-hydroxylase | -1.103012 |
| 66098 | NM_001167736,NM_025351 | CHCHD6 | coiled-coil-helix-coiled-coil-helix domain containing 6 | 1.257914 |
| 66722 | NM_001271533,NM_025728,NM_029160 | SPAG16 | sperm associated antigen 16 | 1.317664 |
| 66840 | NM_025793 | WDR45B | WD repeat domain 45B | 1.164662 |
| 67245 | NM_023324 | PELI1 | pellino 1 | -1.070626 |
| 67574 | NM_026247,NR_037145 | ALG13 | asparagine-linked glycosylation 13 | -1.095571 |
| 67703 | NM_001190911,NM_001190912,NM_001190913,NM_001190914,NM_026324 | KIRREL3 | kin of IRRE like 3 (Drosophila) | 1.159617 |
| 67956 | NM_001310723,NM_001310725,NM_001310727,NM_030241 | KMT5A | lysine methyltransferase 5A | -1.182686 |
| 68545 | NM_001033141 | ECSCR | endothelial cell surface expressed chemotaxis and apoptosis regulator | -1.108138 |
| 68790 | NR_045471,NR_130109 | FENDRR | Foxf1 adjacent non-coding developmental regulatory RNA | -1.050191 |
| 69562 | NM_001081058,NM_027118 | CDK13 | cyclin-dependent kinase 13 | -1.095100 |
| 69940 | NM_001289770,NM_001289771,NM_027270 | EXOC1 | exocyst complex component 1 | -1.001423 |
| 70028 | NM_026700,NM_027293 | DOPEY2 | dopey family member 2 | -1.091254 |
| 70615 | NM_027480 | ANKRD24 | ankyrin repeat domain 24 | 1.132755 |
| 70727 | NM_001316749,NM_027526 | RASGEF1A | RasGEF domain family, member 1A | -1.084790 |
| 71146 | NM_001141983,NM_027694 | GOLGA7B | golgi autoantigen, golgin subfamily a, 7B | -1.038982 |
| 71682 | NM_175173 | WDR27 | WD repeat domain 27 | 1.141001 |
| 72486 | NM_026047 | RNF219 | ring finger protein 219 | 1.136551 |
| 72615 | NM_028301 | ANKS3 | ankyrin repeat and sterile alpha motif domain containing 3 | 1.146357 |
| 74374 | NM_001204229,NM_177562 | CLEC16A | C-type lectin domain family 16, member A | 1.051742 |
| 74513 | NM_001081324 | NETO2 | neuropilin (NRP) and tolloid (TLL)-like 2 | -1.363620 |
| 74589 | NM_001278671 | KBTBD12 | kelch repeat and BTB (POZ) domain containing 12 | 1.015870 |
| 74998 | NM_001033172,NM_001164367 | RAB11FIP2 | RAB11 family interacting protein 2 (class I) | -1.164132 |
| 76740 | NM_133766 | EFR3A | EFR3 homolog A | -1.047971 |
| 77018 | NM_001244952,NM_029838,NM_198711 | COL25A1 | collagen, type XXV, alpha 1 | 1.117674 |
| 77087 | NM_001081379,NR_037865 | ANKRD11 | ankyrin repeat domain 11 | 1.083893 |
| 77938 | NM_001347630,NM_175268,NM_212473 | FAM53B | family with sequence similarity 53, member B | -1.050155 |
| 78808 | NM_001081344 | STXBP5 | syntaxin binding protein 5 (tomosyn) | -1.126562 |
| 80334 | NM_001199242,NM_001199243,NM_001199244,NM_001199245,NM_030265 | KCNIP4 | Kv channel interacting protein 4 | -1.275945 |
| 94092 | NM_053169 | TRIM16 | tripartite motif-containing 16 | -1.046060 |
| 100213 | NM_001037709,NM_199057 | RUSC2 | RUN and SH3 domain containing 2 | 1.026984 |
| 100434 | NM_001159633,NM_133891 | SLC44A1 | solute carrier family 44, member 1 | 1.227851 |
| 102371 | NM_001033208 | MYZAP | myocardial zonula adherens protein | 1.055205 |
| 104103 | NR_002853,NR_027772,NR_027773,NR_027784 | AIM | antisense Igf2r RNA | 1.170999 |
| 105439 | NM_198014 | SLAIN1 | SLAIN motif family, member 1 | -1.055617 |
| 105522 | NM_001024604 | ANKRD28 | ankyrin repeat domain 28 | 1.015562 |
| 106326 | NM_176840 | OSBPL11 | oxysterol binding protein-like 11 | 1.158754 |
| 107993 | NM_001002896 | BFSP2 | beaded filament structural protein 2, phakinin | -1.074515 |
| 108071 | NM_001081414,NM_001143834 | GRM5 | glutamate receptor, metabotropic 5 | -1.321810 |
| 108857 | NM_175375 | ANKHD1 | ankyrin repeat and KH domain containing 1 | 1.020863 |
| 109689 | NM_177231,NM_178220 | ARRB1 | arrestin, beta 1 | 1.066848 |
| 110606 | NM_145927 | FNTB | farnesyltransferase, CAAX box, beta | 1.052533 |
| 111173 | NM_053204,NM_178085,NR_104484 | ERC1 | ELKS/RAB6-interacting/CAST family member 1 | -1.015304 |
| 114642 | NM_001079873,NM_054054 | BRDT | bromodomain, testis-specific | -1.029771 |
| 117149 | NM_001177845,NM_001177846,NM_001177847,NM_054096 | TIRAP | toll-interleukin 1 receptor (TIR) domain-containing adaptor protein | -1.030748 |
| 117600 | NM_001081037,NM_001242411 | SRGAP1 | SLIT-ROBO Rho GTPase activating protein 1 | -1.071439 |
| 140904 | NM_021371,NM_181045 | CALN1 | calneuron 1 | 1.017274 |
| 192285 | NM_001109690,NM_001109691,NM_001346704,NM_138755 | PHF21A | PHD finger protein 21A | -1.096222 |
| 195018 | NM_001045536 | ZZEF1 | zinc finger, ZZ-type with EF hand domain 1 | 1.104156 |
| 207181 | NM_001172121,NM_001172122,NM_001172123,NM_001172124,NM_001172126,NM_178660 | RBMS3 | RNA binding motif, single stranded interacting protein | -1.140900 |
| 208043 | NM_001040398 | SETD1B | SET domain containing 1B | 1.012794 |
| 208727 | NM_207225 | HDAC4 | histone deacetylase 4 | -1.004668 |
| 210417 | NM_172485 | THSD7B | thrombospondin, type I, domain containing 7B | 1.173503 |
| 210710 | NM_181584 | GAB3 | growth factor receptor bound protein 2-associated protein 3 | 1.047263 |
| 210801 | NM_001347498,NM_001347499,NM_001347500,NM_001347501,NM_153135 | UNC5D | unc-5 netrin receptor D | 1.028589 |
| 211147 | NM_001310774,NM_177597 | MARCH11 | membrane-associated ring finger (C3HC4) 11 | 1.028531 |
| 213988 | NM_144812,NM_177124 | TNRC6B | trinucleotide repeat containing 6b | -1.044583 |
| 214058 | NM_001134399,NM_172522 | MEGF11 | multiple EGF-like-domains 11 | 1.038328 |
| 214812 | NM_172536 | ZFP609 | zinc finger protein 609 | -1.002032 |
| 215384 | NM_001122603 | FCGBP | Fc fragment of IgG binding protein | 1.008905 |
| 216345 | NM_001033261 | ZFC3H1 | zinc finger, C3H1-type containing | -1.454050 |
| 216848 | NM_146019 | CHD3 | chromodomain helicase DNA binding protein 3 | 1.212972 |
| 217593 | NM_001167976,NM_172577 | SLC25A21 | solute carrier family 25 (mitochondrial oxodicarboxylate carrier), member 21 | -1.034704 |
| 223658 | NM_001162489,NM_175457 | MROH1 | maestro heat-like repeat family member 1 | 1.338996 |
| 224344 | NM_198302 | RBM11 | RNA binding motif protein 11 | 1.034329 |
| 226169 | NM_001081214,NM_001346801 | PPRC1 | peroxisome proliferative activated receptor, gamma, coactivator-related 1 | 1.075003 |
| 229776 | NM_001080818,NM_001173553 | CDC14A | CDC14 cell division cycle 14A | -1.149519 |
| 231861 | NM_001122730,NM_178242 | TNRC18 | trinucleotide repeat containing 18 | 1.018913 |
| 232035 | NM_001164316,NM_183310 | CCSER1 | coiled-coil serine rich 1 | 1.061246 |
| 239618 | NM_001164593,NM_001164594 | PDZRN4 | PDZ domain containing RING finger 4 | 1.243295 |
| 243931 | NM_172298 | TSHZ3 | teashirt zinc finger family member 3 | -1.106136 |
| 244667 | NM_174853,NM_174854 | DISC1 | disrupted in schizophrenia 1 | 1.065130 |
| 245666 | NM_001005475,NM_001114664 | IQSEC2 | IQ motif and Sec7 domain 2 | -1.096620 |
| 246179 | NM_139309 | FKTN | fukutin | -1.099636 |
| 252864 | NM_001159376,NM_145744 | DUSP15 | dual specificity phosphatase-like 15 | -1.059377 |
| 268977 | NM_001331233,NM_001331234,NM_001331235,NM_001331236,NM_001331237,NM_019919,NM_206958 | LTBP1 | latent transforming growth factor beta binding protein 1 | -1.090287 |
| 269800 | NM_001252083,NM_001347452,NM_175557 | ZFP384 | zinc finger protein 384 | -1.045087 |
| 270028 | NM_001347127,NM_173446 | FAM155A | family with sequence similarity 155, member A | -1.145354 |
| 270685 | NM_001170785,NM_001170786,NM_172308 | MTHFD1L | methylenetetrahydrofolate dehydrogenase (NADP+ dependent) 1-like | 1.077694 |
| 279653 | NM_001105245,NM_001105246 | PCDH19 | protocadherin 19 | -1.045179 |
| 319481 | NM_001170742,NM_001170743,NM_176923 | WDR59 | WD repeat domain 59 | 1.201514 |
| 320158 | NM_001277239,NM_177086 | ZMAT4 | zinc finger, matrin type 4 | -1.017167 |
| 320404 | NM_001081175 | ITPKB | inositol 1,4,5-trisphosphate 3-kinase B | 1.123827 |
| 320661 | NM_001081232 | D5Ertd579e | DNA segment, Chr 5, ERATO Doi 579, expressed | 1.038796 |
| 320736 | NM_178791 | VSTM4 | V-set and transmembrane domain containing 4 | -1.076601 |
| 320940 | NM_001001798,NM_001037863 | ATP11C | ATPase, class VI, type 11C | -1.290135 |
| 329540 | NM_001001986,NM_001134300 | NOL4L | nucleolar protein 4-like | -1.200359 |
| 329650 | NM_177855 | MED12L | mediator complex subunit 12-like | -1.175876 |
| 329679 | NM_001162999 | FNIP2 | folliculin interacting protein 2 | -1.066474 |
| 329908 | NM_183225 | USP24 | ubiquitin specific peptidase 24 | -1.025038 |
| 330941 | NM_001286641,NM_178906 | AI593442 | expressed sequence AI593442 | -1.261427 |
| 333050 | NM_001312914 | KSR2 | kinase suppressor of ras 2 | 1.270264 |
| 380664 | NM_001081193 | LEMD3 | LEM domain containing 3 | -1.216362 |
| 382253 | NM_001024624 | CDKL5 | cyclin-dependent kinase-like 5 | -1.078600 |
| 382406 | NM_027740 | POC1B | POC1 centriolar protein B | -1.022547 |
| 433323 | NM_001004173 | SGPP2 | sphingosine-1-phosphate phosphotase 2 | -1.033897 |
| 100038713 | NR_045741 | GM10432 | predicted gene 10432 | -1.027539 |
| 100101919 | NM_001310335 | DNAH7C | dynein, axonemal, heavy chain 7C | 1.060741 |
| 100503185 | NM_001255991 | BTBD8 | BTB (POZ) domain containing 8 | -1.178220 |

This table lists potential off-target sites which identified by the Cas9-OFFinder algorithm.
