# Supplementary material for: DNA double-strand break-free CRISPR interference delays Huntington’s disease progression in mice
Source: Commun Biol. 2023 Apr 28;6:466. doi: 10.1038/s42003-023-04829-8 (PMC10147674; doi:10.1038/s42003-023-04829-8)
Supplement: Supplementary file 7 — Reporting Summary [file 42003_2023_4829_MOESM7_ESM.pdf]

nature portfolio

Corresponding author(s): DBPR COMMSBIO-22-0171C

Last updated by author(s): Mar 14, 2023

Reporting Summary

Nature Portfolio wishes to improve the reproducibility of the work that we publish. This form provides structure for consistency and transparency in reporting. For further information on Nature Portfolio policies, see our Editorial Policies and the Editorial Policy Checklist.

Statistics

For all statistical analyses, confirm that the following items are present in the figure legend, table legend, main text, or Methods section.

n/a	Confirmed	
☐	☒	The exact sample size (*n*) for each experimental group/condition, given as a discrete number and unit of measurement
☐	☒	A statement on whether measurements were taken from distinct samples or whether the same sample was measured repeatedly
☐	☒	The statistical test(s) used AND whether they are one- or two-sided<br>*Only common tests should be described solely by name; describe more complex techniques in the Methods section.*
☒	☐	A description of all covariates tested
☐	☒	A description of any assumptions or corrections, such as tests of normality and adjustment for multiple comparisons
☐	☒	A full description of the statistical parameters including central tendency (e.g. means) or other basic estimates (e.g. regression coefficient) AND variation (e.g. standard deviation) or associated estimates of uncertainty (e.g. confidence intervals)
☐	☒	For null hypothesis testing, the test statistic (e.g. *F*, *t*, *r*) with confidence intervals, effect sizes, degrees of freedom and *P* value noted<br>*Give P values as exact values whenever suitable.*
☒	☐	For Bayesian analysis, information on the choice of priors and Markov chain Monte Carlo settings
☐	☒	For hierarchical and complex designs, identification of the appropriate level for tests and full reporting of outcomes
☐	☒	Estimates of effect sizes (e.g. Cohen's *d*, Pearson's *r*), indicating how they were calculated

Our web collection on statistics for biologists contains articles on many of the points above.

Software and code

Policy information about availability of computer code

Data collection	No software was used to collect the data.
Data analysis	All data was statistically analyzed using SPSS version 26.0 (IBM corporation), and all graphing was expressed using Prism version 9.0 (GraphPad software) in this study. Western blot bands were quantified using Multi Gauge version3.0 (Fujifilm). Immunohistochemistry was analyzed using ZEN Imaging Software (Blue edition, Zeiss). Locomotor activities of HD mice were tracked and analyzed using Smart Vision 2.5.21 (Panlab).

For manuscripts utilizing custom algorithms or software that are central to the research but not yet described in published literature, software must be made available to editors and reviewers. We strongly encourage code deposition in a community repository (e.g. GitHub). See the Nature Portfolio guidelines for submitting code & software for further information.

Data

Policy information about availability of data

All manuscripts must include a data availability statement. This statement should provide the following information, where applicable:

- Accession codes, unique identifiers, or web links for publicly available datasets
- A description of any restrictions on data availability
- For clinical datasets or third party data, please ensure that the statement adheres to our policy

There are no restriction for any materials used in this study.

Human research participants

Policy information about studies involving human research participants and Sex and Gender in Research.

Reporting on sex and gender	NA
Population characteristics	NA
Recruitment	NA
Ethics oversight	NA

Note that full information on the approval of the study protocol must also be provided in the manuscript.

Field-specific reporting

Please select the one below that is the best fit for your research. If you are not sure, read the appropriate sections before making your selection.

☒ Life sciences ☐ Behavioural & social sciences ☐ Ecological, evolutionary & environmental sciences

For a reference copy of the document with all sections, see nature.com/documents/nr-reporting-summary-flat.pdf

Life sciences study design

All studies must disclose on these points even when the disclosure is negative.

Sample size	The sample size to determine the number of mice and immunohistochemistry analysis was based upon power calculations that take into account sufficient effect size (f-value and cohen's d) and variance from previous publications.
Data exclusions	No data was excluded from analysis. If histology slides were folded or torn, they were excluded from the histological analysis in the mouse brain.
Replication	Findings were not replicated.
Randomization	All animals within a group were randomized and assigned each group.
Blinding	Group allocation was blinded at baseline. In immunohistochemistry analysis, four regions were randomly selected and analyzed by an automatic analysis program. All behavior tests and histological assessments were analyzed via a blind method.

Reporting for specific materials, systems and methods

We require information from authors about some types of materials, experimental systems and methods used in many studies. Here, indicate whether each material, system or method listed is relevant to your study. If you are not sure if a list item applies to your research, read the appropriate section before selecting a response.

Materials & experimental systems

n/a	Involved in the study
☐	☒ Antibodies
☐	☒ Eukaryotic cell lines
☒	☐ Palaeontology and archaeology
☐	☒ Animals and other organisms
☒	☐ Clinical data
☒	☐ Dual use research of concern

Methods

n/a	Involved in the study
☒	☐ ChIP-seq
☐	☒ Flow cytometry
☒	☐ MRI-based neuroimaging

Antibodies

Antibodies used	Anti-SpCas9 (Clone 7A9-3A3) (Mouse monoclonal): Abcam #191468. Lot #. GR3189443-2. 1:1000 dilution for WB. 1:200 for IHC.<br>Anti-Polyglutamine expansion (Clone 5TF1-1C2) (Mouse monoclonal): Millipore #MAB1574. Lot #3091732. 1:1000 dilution for WB.<br>Anti-Huntingtin (Clone 4C8) (Rabbit monoclonal): Millipore #MAB2166. Lot #3850451. 1:1000 dilution for WB.<br>Anti-Huntingtin (Clone mEM48) (Mouse monoclonal): Millipore #MAB5374. Lot #2769369. 1:400 dilution for IHC.<br>Anti-Huntingtin (Clone EPR5526) (Rabbit monoclonal): Abcam #Ab109115. Lot #GR3187604-2. 1:1000 dilution for WB.

	Anti-DARPP-32 (Clone 19A3) (Rabbit monoclonal): Cell signaling technology #2306S. Lot #7. 1:1000 dilution for WB.<br>Anti-NeuN (Clone A60) (Mouse monoclonal): Millipore #MAB377. Lot #324801. 1:2000 for IHC. 1:200 for WB.<br>Anti-Tubulin beta 3 (Mouse monoclonal): BioLegend #801201. Lot #B264428. 1:400 for IHC.<br>Anti-BDNF (Clone EPR1292) (Rabbit monoclonal): Abcam #Ab108319. Lot #GR3227037-4. 1:1000 for WB. 1:200 for IHC.<br>Anti-beta-Actin (Clone C4) (Mouse monoclonal): Santa Cruz Biotechnology #SC-47778. Lot #C1919. 1:2000 for WB.<br>Anit-mouse IgGk BP-HRP (Mouse monoclonal): Santa Cruz Biotechnology #SC-516102. Lot #B0519. 1:3000 for WB.<br>Anit-rabbit IgG-HRP (Rabbit monoclonal): Santa Cruz Biotechnology #SC-2357. Lot #L1218. 1:3000 for WB.<br>Anti-rabbit IgG (H+L) Cross-Adsorbed Secondary Antibody, Alexa Flor 594 (Rabbit polyclonal): Invitrogen #Ab150080. Lot #GR3232361. 1:400 for IHC.<br>Anti-mouse IgG (H+L) Cross-Adsorbed Secondary Antibody, Alexa Flor 594 (Mouse polyclonal): Invitrogen #A11005. Lot #2043369. 1:400 for IHC.<br>Anti-rabbit IgG (H+L) Cross-Adsorbed Secondary Antibody, Alexa Flor 488 (Rabbit polyclonal): Invitrogen #A11008. Lot #2140660. 1:400 for IHC.<br>Anti-mouse IgG (H+L) Cross-Adsorbed Secondary Antibody, Alexa Flor 488 (Mouse polyclonal): Invitrogen #A11001. Lot #2051237. 1:400 for IHC.
Validation	According to the manufacturer's website, the SpCas9 antibody is recommended for detection of SpCas9 of streptococcus pyogenes origin by Western blot.<br>According to the manufacturer's website, Polyglutamine expansion (Clone 5TF1-1C2) antibody is recommended for detection of human origin by Western blot.<br>According to the manufacturer's website, Huntingtin (Clone 4C8), Huntingtin (Clone mEM48) and Huntingtin (Clone EPR5526) antibodies are recommended for detection of human and mouse origin by Western blot or IHC.<br>According to the manufacturer's website, DARPP-32 (Clone 19A3), NeuN (Clone A60) and BDNF (Clone EPR1292) antibodies are recommended for detection of mouse origin by Western blot.<br>According to the manufacturer's website, Tubulin beta 3, NeuN (Clone A60), BDNF (Clone EPR1292), and GFAP antibodies are recommended for detection of mouse origin by IHC.<br>In case of Tubulin beta 3, according to the manufacturer's website, the Tubulin beta 3 antibody is recommended for detection of Tubulin beta 3 of Human, mouse and rat origin by WB, ICC, IHC-P and FC; See relevant citation for IHC-Fr, β-Catenin signals regulate cell growth and the balance between progenitor cell expansion and differentiation in the nervous system. Zechner D., et al. 2003.

Eukaryotic cell lines

Policy information about cell lines and Sex and Gender in Research

Cell line source(s)	Six human HD fibroblast lines were purchased from Coriell Institute (catalog #GM09197, #GM21756, #GM04022, #GM04855, GM04775, and #GM07492). HEK293T cell line was purchased from Korean Collection for Type Cultures (catalog #CRL-11268).
Authentication	These cells were authenticated by the cell center, but not reauthenticated by our laboratory.
Mycoplasma contamination	These cells were tested by the cell center and confirmed that there was no mycoplasma contamination, but not retested by our laboratory.
Commonly misidentified lines (See ICLAC register)	None

Animals and other research organisms

Policy information about studies involving animals; ARRIVE guidelines recommended for reporting animal research, and Sex and Gender in Research

Laboratory animals	All mice were on the B6CBAF1 background strain and carried human HD gene that includes 160 ± 5 CAG repeats.<br>They were received stereotaxic surgery at 4 weeks of age and sacrificed at 13 weeks of age.
Wild animals	This study did not involve wild animals.
Reporting on sex	Both female and male were used in this study and randomly assigned experimental groups.
Field-collected samples	This study did not involve samples collected from the field.
Ethics oversight	The Institutional Animal Care and Use Committee (IACUC) of the Yonsei University College of Medicine

Note that full information on the approval of the study protocol must also be provided in the manuscript.

Flow Cytometry

Plots

Confirm that:

☒ The axis labels state the marker and fluorochrome used (e.g. CD4-FITC).

☒ The axis scales are clearly visible. Include numbers along axes only for bottom left plot of group (a 'group' is an analysis of identical markers).

☒ All plots are contour plots with outliers or pseudocolor plots.

☒ A numerical value for number of cells or percentage (with statistics) is provided.

Methodology

Sample preparation	Three days after transfection of plasmids encoding Cas9 and sgRNA (single guide RNA) and reporter plasmids, adherent 293T cells were trypsinized and resuspended in 2% FBS in PBS. Single-cell suspensions were flow cytometrically analyzed and sorted.<br>Untransfected cells and cells transfected with either mRFP vector alone or eGFP vector alone were used as controls.
Instrument	FACSAria II cell sorter (BD Biosciences, San Jose, CA, USA)
Software	Flow cytometry data were analyzed with FlowJo v10.
Cell population abundance	In the experiment groups, transfected mRFP+ cells comprised approximatedly ~20-50% of the total cell populations, depending on the sample. The percent of the cells positive for both mRFP and eGFP (i.e., mRFP+ and eGFP+) were variable depending on the indel-generating efficiency of the corresponding single guide RNA.
Gating strategy	Cells were gated using forward/side scatter parameters to exclude small debris and then gated on forward scatter height vs. width to remove cell doublets.

☒ Tick this box to confirm that a figure exemplifying the gating strategy is provided in the Supplementary Information.
